# Important Medical Works for Publication

**Published:** 1878-11

**Authors:** 


MR. HENRY C. LEA calls attention to the following important works in preparation for early publication.

*The National Dispensatory.* Containing the Natural History, Chemistry, Pharmacy, Actions and Uses of Medicines, and including the preparations of the Pharmacopœas of the United States and Great Britain. By Alfred Stillé, M. D., L. L. D., Prof. of Theory and Practice of Med., and of Clinical Med. in the University of Pennsylvania, etc., and John M. Maisch, Ph. D., Prof. of Mat. Med. and Bot. in the Phil. Col. of Pharmacy, Secretary of the Am. Pharm. Association. With 205 illustrations. In one handsome octavo volume of over 1400 pages.

*Clinical Manual for the Study of Medical Cases:* For the use of Students and Practitioners of Medicine. By James Finlayson, M. D., Phys. and Lect. on Clin. Med. in the Glasgow Western Infir., etc. In one handsome 12mo. vol. of about 500 pp., with 85 illustrations.

*The Principles and Practice of Surgery.* By John Ashhurst, Jr., M. D., Prof. of Clin. Surg., of Pa.; Surgeon to the Episc. Hosp, Phila. Second and Revised Edition. In one large and handsome 8vo. vol. of about 1000 pages, with about 550 illustrations.

*The Principles and Practice of Gynæcology*, for the Use of Students and Practitioners of Medicine. By Thomas Addis Emmet, M. D., Surgeon to the Woman's Hospital, New York, etc. In one handsome octavo volume of over 800 pages, with numerous illustrations.

*The Practice of Surgery.* By Thomas Bryant, F. R. C. S., Surgeon to Guy's Hospital. Second American, from the Second and revised English Edition. In one large and very handsome imperial octavo volume of over 1000 pages, with about six hundred engravings on wood.

*A System of Human Anatomy*: Including its Medical and Surgical Relations. For the Use of Practitioners and Students of Medicine. By Harrison Allen, M. D., Prof. of Physiology in the Univ. of Pa. With an Introductory Chapter on Histology, by E. O. Shakespeare, M. D., Ophthalmologist to the Phila. Hospital. In one large and handsome quarto volume, with several hundred original illustrations on lithographic plates, and numerous wood-cuts in the text.

*Manual of Pathological Histology.* By V. Cornil, Prof. in the Faculty of

Medicine, Paris, and L. Ranvier, Prof. in the College of France. Translated, with Notes and Additions, by E. O. Shakespeare, M. D., Pathologist and Ophthalmic Surgeon to Philada. Hospital, Lecturer on Refraction and Operative Ophthalmic Surgery in Univ. of Pa. In one very handsome octavo volume of about 600 pages, with over 300 illustrations.

